# Oxidative balance score is independently associated with reduced prevalence of sarcopenia among US adults with metabolic syndrome

**DOI:** 10.3389/fnut.2025.1529140

**Published:** 2025-04-08

**Authors:** Miaohong Wang, Huan Shi

**Affiliations:** ^1^Health Management Center, The Third Affiliated Hospital of Soochow University, Changzhou, China; ^2^Department of Endocrinology, The Third Affiliated Hospital of Soochow University, Changzhou, China

**Keywords:** oxidative balance score, oxidative stress, sarcopenia, metabolic syndrome, NHANES

## Abstract

**Background:**

This research seeks to explore the link between the oxidative balance score (OBS) and sarcopenia in American adults with Metabolic Syndrome (MetS) using data from a national, population-based survey.

**Methods:**

The study included 3,625 participants diagnosed with Metabolic Syndrome, all aged 20 years and above, derived from NHANES datasets spanning 1999–2006 and 2011–2018. OBS evaluation was based on 16 dietary and 4 lifestyle elements. MetS diagnosis followed the NCEP-ATP III guidelines, while sarcopenia identification was based on FNIH standards. We employed multivariate logistic regression analyses to delve into the connections between OBS and sarcopenia within the MetS cohort.

**Results:**

Sarcopenia was found in 17.46% of the participants. In models adjusted for all variables, OBS, dietary OBS, and lifestyle OBS each showed a significant inverse relationship with sarcopenia among MetS individuals [OBS: OR = 0.959, 95%CI: (0.948, 0.982), *P* trend = 0.0005; dietary OBS: OR = 0.963, 95%CI: (0.939, 0.989), *P* trend = 0.0055; lifestyle OBS: OR = 0.860, 95%CI: (0.787, 0.939), *P* trend = 0.0011]. Higher scores in OBS were consistently linked with a decreased incidence of sarcopenia (all *P* for trend < 0.05). Restricted cubic spline analysis confirmed that these relationships were linear. The impact of age was significant, with OBS benefits only observed in those aged 40 and older.

**Conclusions:**

Maintaining a diet and lifestyle rich in antioxidants is both independently and collectively linked with a lower occurrence of sarcopenia in individuals with MetS. These results bolster the proposition of developing OBS-centered preventive strategies for sarcopenia in MetS patients, particularly those aged 40 years and older.

## 1 Introduction

Sarcopenia is a common condition affecting skeletal muscle associated with aging, marked by a steady, extensive reduction in muscle mass and strength (1, 2). It represents a critical aspect of elderly frailty and significantly affects elderly health and wellbeing (3). The occurrence of sarcopenia varies according to its defined criteria, gender, and age, impacting about 10–16% of the elderly population (4). Furthermore, sarcopenia's incidence is notably higher among individuals with chronic non-communicable diseases such as cardiovascular disease (CVD), liver disorders, chronic obstructive pulmonary disease, chronic kidney disease, and cancer (5–7). On its own, sarcopenia is associated with various adverse health effects, including cognitive decline, fractures, falls, hospitalizations, and heightened mortality risk (6, 8). Though primarily seen as a condition of old age, emerging research points to its significant relevance in younger adults as well (1, 9).

Metabolic syndrome (MetS) encompasses a set of cardiometabolic risk factors that include insulin resistance, central obesity, dyslipidemia, and hypertension (10). MetS poses a significant global public health issue, affecting roughly a quarter of the global adult population and imposing substantial economic costs annually (11, 12). While not a disease in itself, extensive epidemiological data indicate that MetS is closely linked to various severe health conditions, such as CVD, cancer, chronic kidney disease, and osteoarthritic disorders (13–15). There is growing evidence that MetS is closely related to the onset of sarcopenia, which is becoming a focal point of scientific inquiry (16). Clinical research suggests that sarcopenia related to MetS might be connected to poor clinical outcomes (17, 18).

Oxidative stress serves as a common pathogenic factor for both MetS and sarcopenia, potentially mediating the interaction between muscle degradation and metabolic imbalance (19). The oxidative balance score (OBS) is a novel metric for gauging an individual's combined exposure to pro-oxidants and antioxidants. OBS offers a refined evaluation of an individual's intricate oxidative balance by incorporating both dietary and lifestyle sources of antioxidants and pro-oxidants (20). Prior observational research has linked OBS with reduced muscle mass (21–23). Nevertheless, the protective role of OBS against sarcopenia development within the MetS group, especially its potential benefit for early-onset sarcopenia (20–39 years of age) remains under-researched. Exploring this relationship could have significant public health benefits, as controlling OBS might help prevent sarcopenia in the MetS demographic and alleviate its associated health burdens.

This research employed data from the National Health and Nutrition Examination Survey (NHANES), a nationally representative, population-based cross-sectional study, to explore how OBS influences the development of sarcopenia among adults with Metabolic Syndrome (MetS). These insights suggest that maintaining an antioxidant-rich diet and lifestyle could help thwart sarcopenia in individuals with MetS.

## 2 Materials and methods

### 2.1 Study design and population

NHANES is the principal survey conducted by the National Center for Health and Statistics (NCHS) to thoroughly evaluate the health and nutritional status of non-institutionalized U.S. children and adults and to generate vital health data. Since 1999, NHANES has operated biennially, randomly selecting about 5,000 representative samples annually from across the U.S., making it a continuous, serial cross-sectional study with a sophisticated, multistage, probability sampling framework. NHANES involves demographic and health questionnaires and a series of physical exams. All NHANES protocols are approved by the NCHS Ethics Review Board, and all participants provided written informed consent.

**Figure 1:**
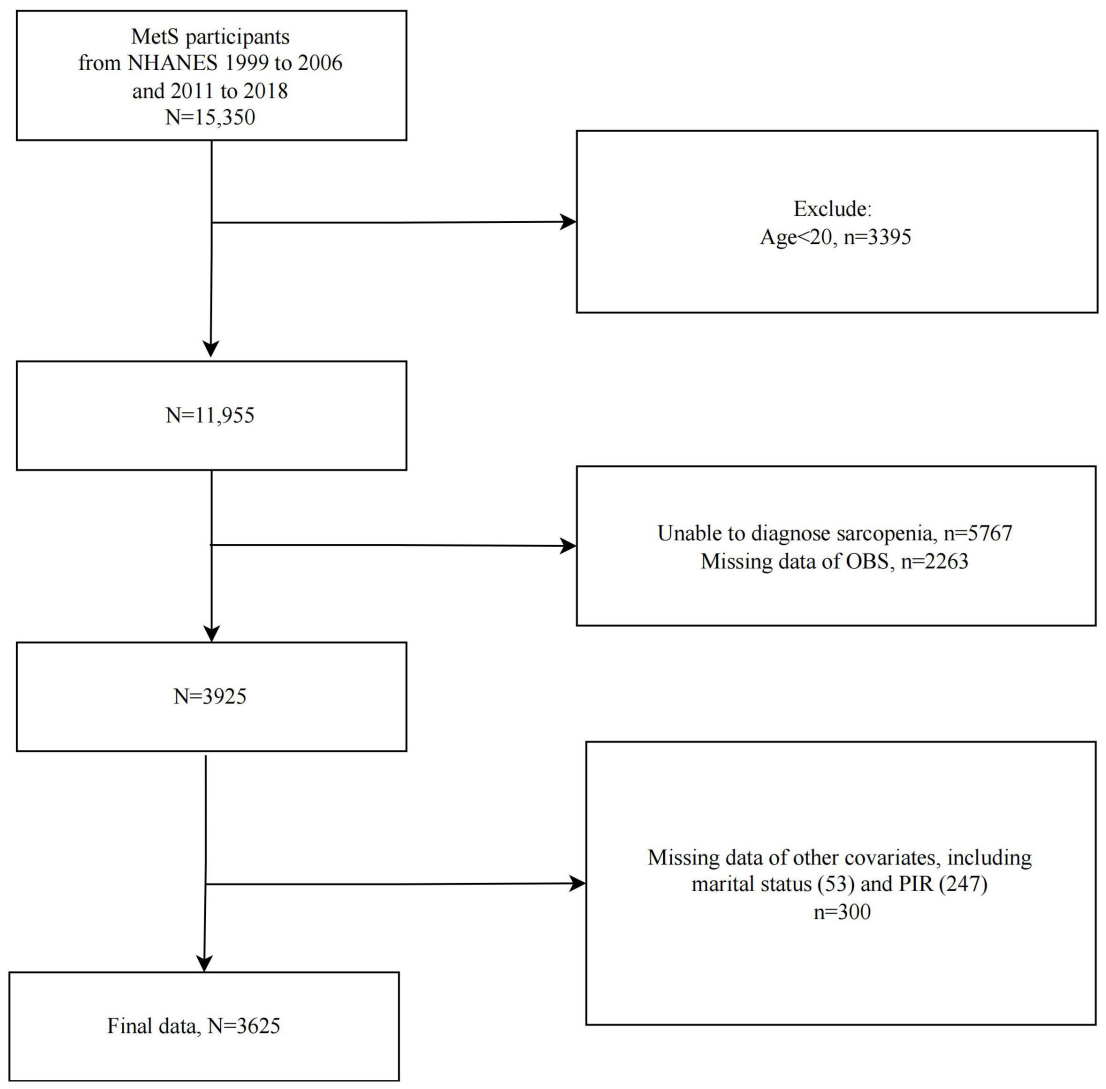
Flowchart of study population selection, NHANES 1999–2006 and 2011–2018. MetS, Metabolic Syndrome; OBS, oxidative balance score.

We included 15,350 MetS participants from eight cycles of NHANES spanning 1999–2006 and 2011–2018, and subsequently excluded those under 20 years old (*n* = 3,395), those unable to be diagnosed with sarcopenia (*n* = 5,767), those lacking OBS data (*n* = 2,263), and those missing other covariates (*n* = 300). Ultimately, 3,625 eligible adults with MetS were included in the study (Figure 1).

### 2.2 Evaluation of OBS

We utilized a validated method from prior research for evaluating the Oxidative Balance Score (OBS) that have been published in the literature (21, 24). In brief, the OBS incorporates 16 dietary elements and 4 lifestyle factors. The dietary OBS extensively measures intake of dietary fiber, carotenoids, riboflavin, niacin, vitamin B6, total folate, vitamin B12, vitamin C, vitamin E, calcium, magnesium, zinc, copper, selenium, total fat, and iron. Lifestyle OBS factors include physical activity, body mass index (BMI), alcohol intake, and serum cotinine levels. Dietary data was sourced from the average of two 24-h dietary recalls, consisting of an initial in-person dietary interview at a mobile examination center and a subsequent telephone follow-up 3–10 days later. Physical activity was quantified using metabolic equivalents (25), calculated from the frequency and duration of activities related to work, leisure, and transport, along with their respective MET values. BMI was calculated by dividing weight in kilograms by the square of height in meters. Serum cotinine, a primary nicotine metabolite, indicated smoking exposure. Antioxidants were scored as 2, 1, or 0 based on gender-specific benchmarks from high to low, while pro-oxidants were scored inversely. Alcohol consumption categories on the dietary recall were scored differently for men and women; those consuming over 30/15 g/d received a score of 0, while 0–30/0–15 g/d and no alcohol intake were scored 1 and 2, respectively (Supplementary Table S1). Each participant's overall OBS, dietary OBS, and lifestyle OBS scores were computed based on these criteria.

### 2.3 Diagnosis of MetS

The National Cholesterol Education Program-Adult Treatment Panel III guidelines for diagnosing MetS are well-established in NHANES-based research (24, 26). MetS was identified through a combination of central obesity, elevated blood sugar, abnormal blood lipid levels, and hypertension. It was defined by meeting three or more of the following criteria: 1. waist circumference (WC) of ≥102 cm for men or ≥88 cm for women; 2. serum triglycerides (TG) levels of ≥150 mg/dL; 3. serum high-density lipoprotein cholesterol (HDL-C) levels of < 40 mg/dL in men or < 50 mg/dL in women; 4. fasting blood glucose (FBG) of ≥100 mg/dL or on diabetes medication; 5. blood pressure (BP) of ≥130/85 mm Hg. Data regarding serum TG, HDL-C, and FBG were extracted from NHANES biochemical profiles, while blood pressure readings were acquired from three consecutive tests conducted by trained staff at the mobile examination centers.

### 2.4 Assessment of sarcopenia

We utilized the diagnostic standards from the Foundation for the National Institutes of Health (FNIH) Sarcopenia Project for defining sarcopenia in adults, a method frequently employed in prior NHANES studies (21, 23, 27). Appendicular lean mass (ALM), measured by dual-energy X-ray absorptiometry (DXA), served to evaluate muscle mass in participants. NHANES provided DXA body composition data from 1999–2006 to 2011–2018. Notably, during NHANES 2011–2018, DXA assessments were available only for individuals aged 8–59 years, while in NHANES 1999–2006, they were accessible for individuals aged 60 years and above (DXA was performed for all ≥8 years of age from 1999 to 2004, and for those aged 8–69 years during 2005–2006) (28). As per NHANES protocol, individual's ineligible for DXA exams due to being taller than 192.5 cm, heavier than 136.4 kg, or pregnant were excluded. The ratio of ALM to BMI was applied to identify sarcopenia, defined as < 0.789 in men or < 0.521 in women (29). Early-onset sarcopenia was characterized as sarcopenia diagnosed in individuals aged 20–39 years (30).

### 2.5 Covariates

Several potential covariates were included based on earlier studies, covering age, sex (male/female), racial/ethnic background (Mexican American/non-Hispanic White/non-Hispanic Black/other Hispanic/other race), educational attainment (< high school/high school/>high school), income-poverty ratio (PIR), marital status (single/non-single), and daily caloric intake (31). Demographic details were sourced from the NHANES demographic files. Daily caloric intake (kcal/day) was obtained from self-reported dietary recalls conducted in person at mobile examination centers.

### 2.6 Statistical analysis

We properly adjusted all analyses in line with the NHANES Analytic Guidelines to reflect the intricate design of the NHANES study. Data manipulation and statistical evaluations were conducted using EmpowerStats (X&Y Solutions, Inc., Boston, MA) and R software (version 4.2.3). A two-tailed *P*-value below 0.05 was deemed to indicate statistical significance. In the initial analysis, participants were categorized based on their sarcopenia status. Continuous measures were reported as mean ± standard error and assessed using a weighted *t*-test, while categorical data were presented as number (percentage) and evaluated using a weighted chi-square test. To examine the distinct relationship of OBS with sarcopenia in MetS subjects, we developed several multivariate logistic regression models, deriving odds ratios (OR) and 95% confidence intervals (CI). The preliminary models were unadjusted, Model 1 incorporated adjustments for age, sex, race, PIR, educational level, and marital status, and Model 2 further included adjustments for dietary energy consumption beyond Model 1. Restricted cubic spline (RCS) and curve smoothing techniques were applied to investigate possible dose-response relationships of OBS with sarcopenia incidence among MetS individuals, utilizing the rms package. Stratified analyses were conducted to assess the consistency of this relationship across different subgroups and to identify potential modifiers through interaction tests. For key effect modifiers, corresponding multivariate logistic regression and RCS analyses were performed within their subgroups. In the sensitivity checks, we employed an alternative widely recognized MetS diagnostic criterion, the International Diabetes Federation (IDF), to confirm the robustness of our findings (25).

## 3 Results

### 3.1 Baseline characteristics

The study included 3,625 MetS participants, averaging 48.905 years old, 51.264% male, and 75.363% non-Hispanic White. Sarcopenia was diagnosed in 633 individuals, representing a prevalence of 17.46%. Compared to those without sarcopenia, those diagnosed with the condition tended to be older, had lower PIR and energy consumption, and were more likely to be Mexican American/Other Hispanic/Other ethnicity and have ≤ high school education. Notably, those with sarcopenia had significantly lower OBS, dietary OBS, and lifestyle OBS (all *P* trend < 0.001; Table 1).

**Table 1:** Baseline analysis according to sarcopenia status among MetS participants, NHANES 1999–2006 and 2011–2018.

**Characteristic**	**Total (*****n*** = **3,625)**		**No-sarcopenia (*****n*** = **2,992)**		**Sarcopenia (*****n*** = **633)**		***P*-value**
	**Mean**	**SD**	**Mean**	**SD**	**Mean**	**SD**	
Age	48.905	0.335	47.990	0.342	54.924	0.735	< 0.0001
PIR	3.063	0.047	3.118	0.048	2.706	0.096	< 0.0001
OBS.dietary	15.846	0.178	16.053	0.199	14.485	0.362	< 0.001
OBS.lifestyle	3.394	0.033	3.410	0.035	3.291	0.080	< 0.001
OBS	19.240	0.193	19.463	0.214	17.776	0.375	< 0.001
Energy intake	2161.829	17.945	2182.668	20.970	2024.759	48.071	0.006
	***n***	**%**	***n***	**%**	***n***	**%**	
**Sex**							0.05
male	1,788	51.264	1,449	50.417	339	56.834	
female	1,837	48.736	1,543	49.583	294	43.166	
**Race**							< 0.0001
Mexican American	733	7.749	502	6.653	231	14.957	
Non-Hispanic Black	544	7.001	518	7.745	26	2.107	
Non-Hispanic White	1,824	75.363	1,540	76.505	284	67.851	
Other Hispanic	227	4.980	172	4.348	55	9.136	
Other race	297	4.907	260	4.749	37	5.950	
**Marital status**							0.596
Non-single	2,474	70.945	2,043	70.738	431	72.312	
Single	1,151	29.055	949	29.262	202	27.688	
**Education**							< 0.0001
< High school	358	4.671	216	3.473	142	12.552	
High school	1,430	40.126	1,164	39.668	266	43.144	
>High school	1,837	55.203	1,612	56.859	225	44.305	

MetS, Metabolic Syndrome; NHANES, the National Health and Nutrition Examination Survey; OBS, oxidative balance score; PIR, income-poverty ratio.

Continuous variables were expressed as mean ± standard error and analyzed using a weighted t-test, whereas categorical variables were expressed as number (percentage) and analyzed using a weighted chi-square test.

### 3.2 Multivariate logistic regression analysis

When OBS was considered as a continuous variable, in the initial analysis and Model 1, OBS was found to significantly inversely correlate with sarcopenia incidence among MetS patients [crude model: OR = 0.965, 95%CI: (0.948, 0.982), *P* trend = 0.0001; Model 1: OR = 0.966, 95%CI: (0.949, 0.984), *P* trend =0.0003]. When adjusting for all variables in Model 2, OBS continued to show an inverse association with sarcopenia among these participants, each additional unit of OBS will reduce the incidence rate of sarcopenia in MetS patients by 4.1% [OR: 0.96 (0.95, 0.97), *P* < 0.001]. When OBS was considered as a categorical variable, the incidence of sarcopenia markedly decreased with higher quartiles of OBS (P trend = 0.0026). Relative to the baseline group (Q1), belonging to Q3 and Q4 of OBS corresponded with a substantially reduced incidence of sarcopenia [Q3: OR = 0.659, 95% CI: (0.458, 0.949), *P* trend = 0.0270; Q4: OR = 0.494, 95% CI: (0.314, 0.776), *P* trend = 0.0028]. Both dietary OBS and lifestyle OBS showed significant and inverse correlations with sarcopenia odds in MetS individuals. Each increment in dietary OBS and lifestyle OBS was linked to a 3.7 and 14% decrease in sarcopenia odds, respectively. Higher levels of dietary OBS and lifestyle OBS were each associated with a notably lower sarcopenia prevalence (*P* trend = 0.0091 and 0.0093, respectively), with Q4 levels of dietary OBS and lifestyle OBS relative to the baseline group showing reductions in sarcopenia prevalence of 51.4 and 40.4%, respectively (Table 2).

**Table 2:** Association of OBS, dietary OBS, and lifestyle OBS with prevalence of sarcopenia in MetS populations, NHANES 1999–2006 and 2011–2018.

**Variables**	**Crude model OR (95%CI) *P*-value**	**Model 1 OR (95%CI) *P*-value**	**Model 2 OR (95%CI) *P*-value**
**OBS**	0.965 (0.948, 0.982) 0.0001	0.966 (0.949, 0.984) 0.0003	0.959 (0.938, 0.981) 0.0005
**OBS quartile**			
Q1	Ref.	Ref.	Ref.
Q2	0.726 (0.505, 1.043) 0.0859	0.714 (0.491, 1.038) 0.0802	0.698 (0.474, 1.027) 0.0707
Q3	0.655 (0.469, 0.915) 0.0145	0.689 (0.484, 0.981) 0.0413	0.659 (0.458, 0.949) 0.0270
Q4	0.518 (0.361, 0.743) 0.0005	0.533 (0.370, 0.767) 0.0010	0.494 (0.314, 0.776) 0.0028
***P*** **for trend**	0.0004	0.0014	0.0026
**OBS. dietary**	0.967 (0.948, 0.985) 0.0006	0.969 (0.951, 0.988) 0.0020	0.963 (0.939, 0.989) 0.0055
**OBS. dietary quartile**			
Q1	Ref.	Ref.	Ref.
Q2	0.714 (0.487, 1.047) 0.0876	0.705 (0.477, 1.043) 0.0831	0.686 (0.454, 1.038) 0.0774
Q3	0.714 (0.496, 1.028) 0.0728	0.766 (0.521, 1.124) 0.1760	0.727 (0.484, 1.092) 0.1272
Q4	0.512 (0.346, 0.756) 0.0010	0.531 (0.358, 0.788) 0.0022	0.486 (0.294, 0.803) 0.0058
***P*** **for trend**	0.0014	0.0045	0.0091
**OBS. lifestyle**	0.880 (0.807, 0.960) 0.0045	0.861 (0.788, 0.941) 0.0013	0.860 (0.787, 0.939) 0.0011
**OBS. lifestyle quartile**			
Q1	Ref.	Ref.	Ref.
Q2	0.796 (0.548, 1.157) 0.2341	0.798 (0.551, 1.156) 0.2358	0.785 (0.543, 1.135) 0.2008
Q3	0.770 (0.529, 1.123) 0.1772	0.761 (0.512, 1.130) 0.1778	0.757 (0.511, 1.123) 0.1698
Q4	0.649 (0.456, 0.925) 0.0183	0.599 (0.416, 0.865) 0.0072	0.596 (0.414, 0.858) 0.0063
***P*** **for trend**	0.0227	0.0097	0.0093

MetS, Metabolic Syndrome; NHANES, the National Health and Nutrition Examination Survey; OBS, oxidative balance score; PIR, income-poverty ratio.

Crude models did not adjust for any covariates, model 1 adjusted for age, sex, race, PIR, educational attainment, and marital status, and model 2 additionally adjusted for dietary energy intake above model 1.

### 3.3 RCS analysis

**Figure 2:**
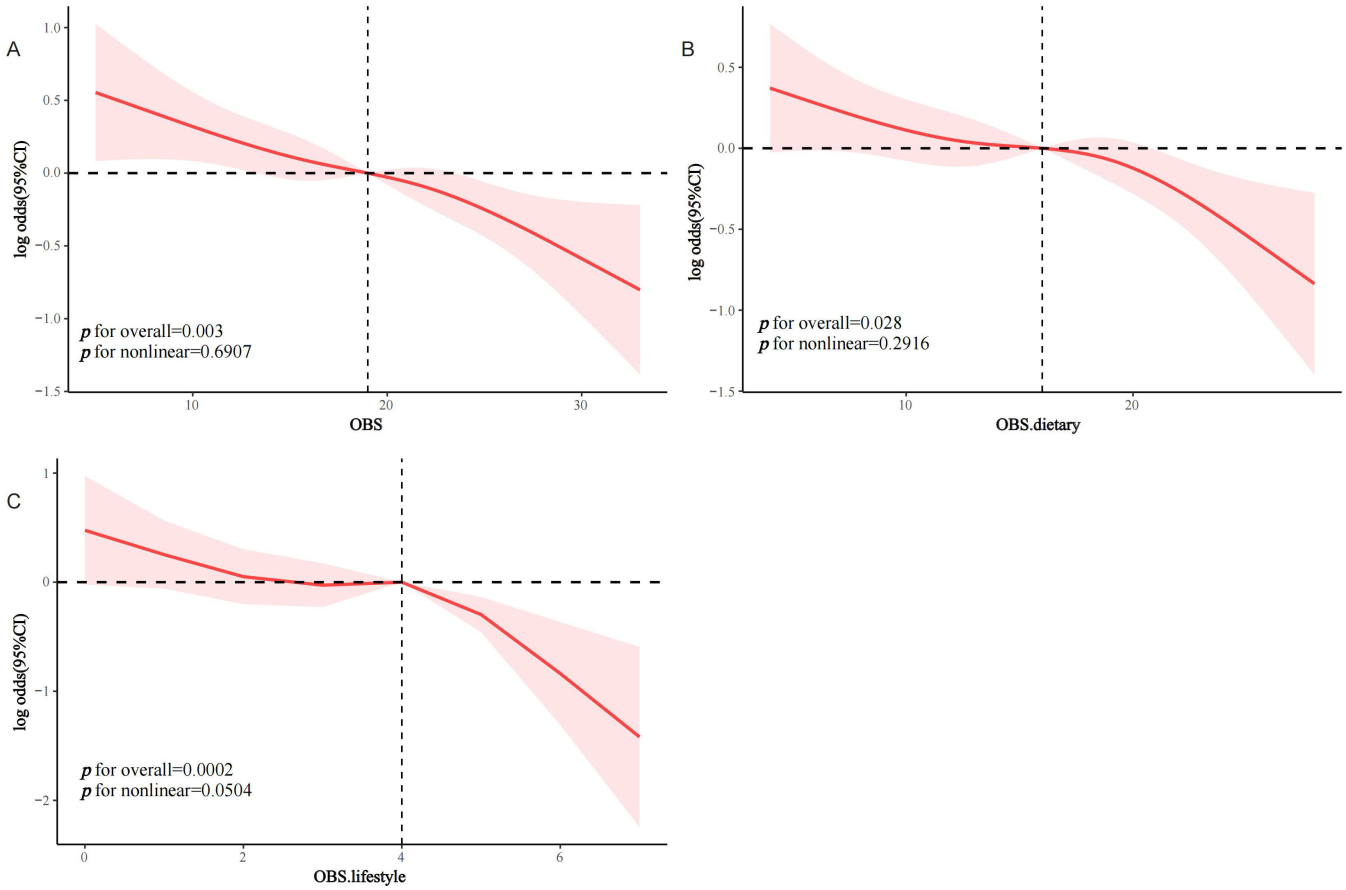
RCS analysis of the association between OBS and the prevalence of sarcopenia in the MetS population. **(A)** OBS; **(B)** dietary OBS; **(C)** lifestyle OBS. MetS, Metabolic Syndrome; OBS, oxidative balance score.

RCS analysis with curve smoothing indicated a clear inverse linear relationship between OBS and sarcopenia occurrence in MetS subjects (*P* trend for overall = 0.003, *P* trend for non-linear = 0.6907; Figure 2A). Similarly, dietary OBS (Figure 2B) and lifestyle OBS (Figure 2C) both had a negative dose-response association with the odds of sarcopenia in MetS individuals (*P* trend for non-linear = 0.2916 and 0.0504, respectively).

### 3.4 Stratified analysis

**Figure 3:**
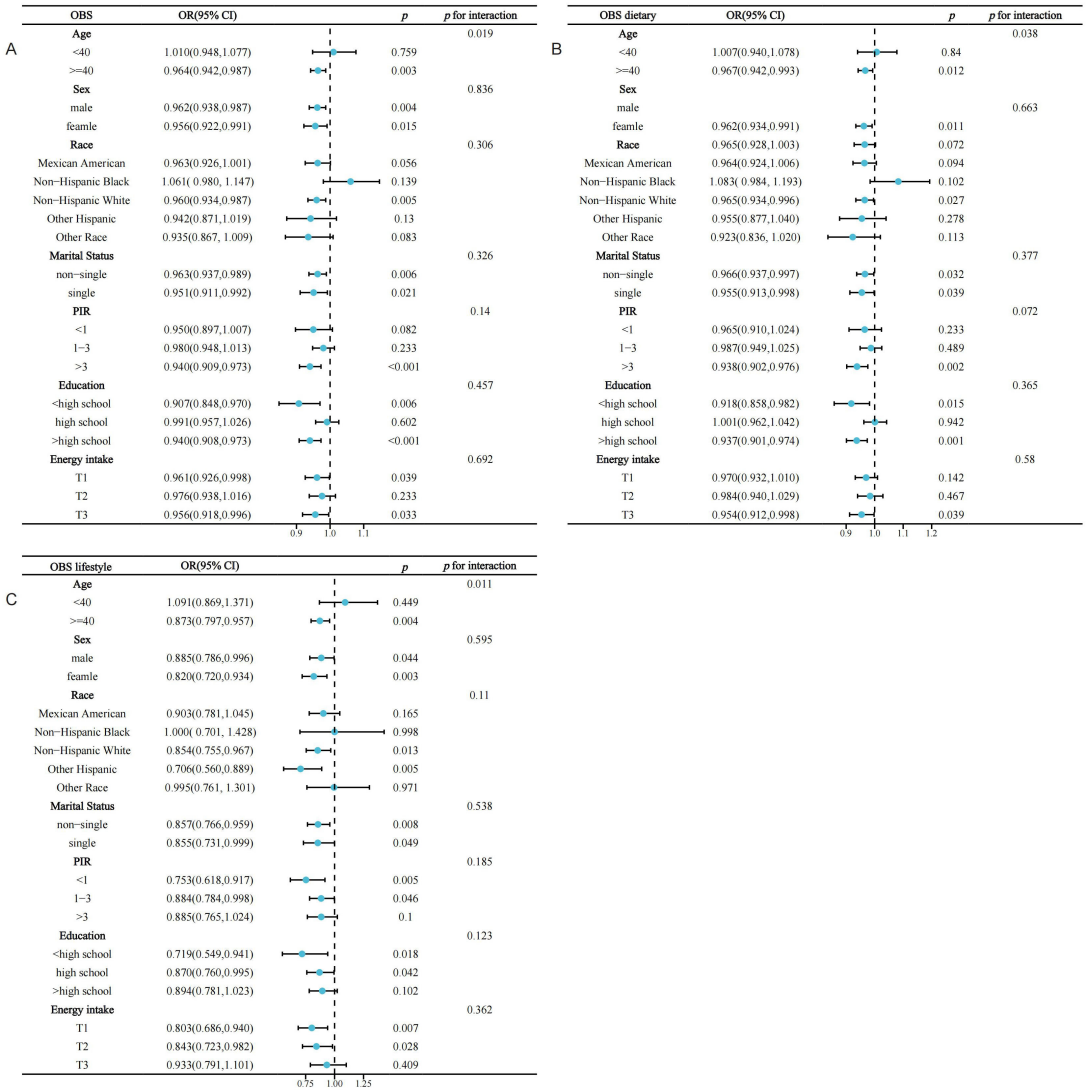
Stratified analysis of the association between OBS and the prevalence of sarcopenia in the MetS population based on included covariates. MetS, Metabolic Syndrome; OBS, oxidative balance score; PIR, income-poverty ratio. **(A)** OBS; **(B)** dietary OBS; **(C)** lifestyle OBS.

**Figure 4:**
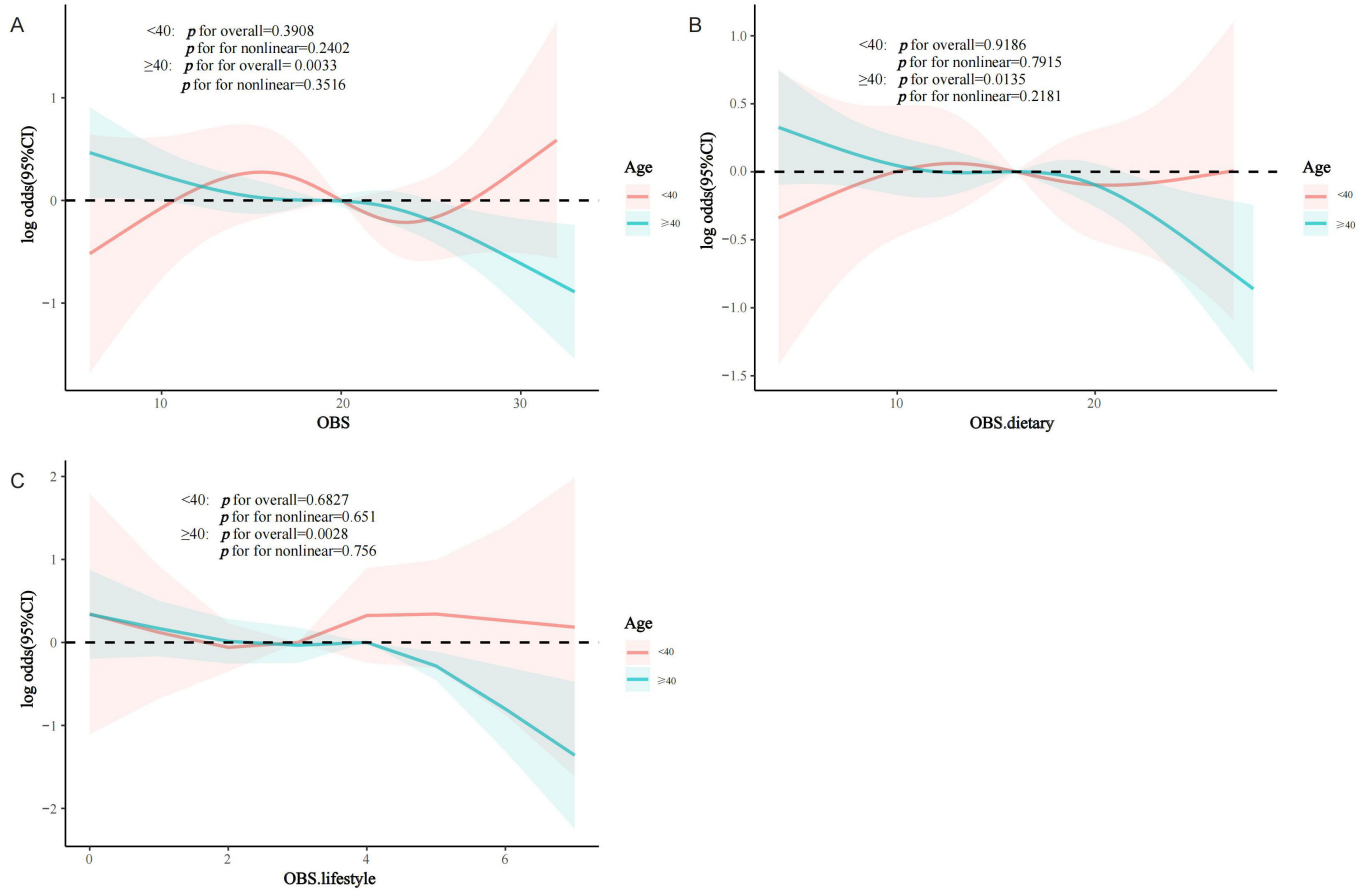
RCS analysis according to age (< 40 years/≥40 years) subgroups. **(A)** OBS; **(B)** dietary OBS; **(C)** lifestyle OBS. MetS, Metabolic Syndrome; OBS, oxidative balance score.

Interaction assessments revealed that the correlations between OBS, dietary OBS, and lifestyle OBS with sarcopenia in the MetS group were consistent across various subgroups. Yet, age proved to be a significant modifying factor (OBS: *P* for interaction = 0.019; dietary OBS: *P* for interaction = 0.038; lifestyle OBS: *P* trend = 0.011). Notably, the relationships of all OBS variants with sarcopenia in those with MetS were evident only in participants aged 40 years and above, with no connections found in younger onset sarcopenia (Figures 3A–C). Subsequent multivariate regression analyses across age groups (20–39 and ≥40 years) also reflected that OBS, dietary OBS, and lifestyle OBS did not correlate with the occurrence of sarcopenia in younger MetS individuals when all confounders were accounted for (all *P* trend > 0.05; Supplementary Table S2). However, in individuals aged 40 years and older, OBS, dietary OBS, and lifestyle OBS all showed a negative association with sarcopenia within the MetS group [OBS: OR = 0.964, 95% CI: (0.942, 0.987), *P* trend = 0.003; dietary OBS: OR = 0.967, 95% CI: (0.942, 0.993), *P* trend = 0.0012; lifestyle OBS: OR = 0.873, 95% CI: (0.797,0.957), *P* trend = 0.0041], with higher levels of each indicating significantly lower sarcopenia rates (*P* for trend 0.0060, 0.0162, and 0.0156, respectively). At the fourth quartile (compared to the first), OBS, dietary OBS, and lifestyle OBS were linked with 49.1, 49.5, and 42.4% decreased odds of sarcopenia, respectively (Table 3). Additional RCS analysis revealed that while OBS, dietary OBS, and lifestyle OBS showed no link with the prevalence of early-onset sarcopenia in MetS, they were all significantly and linearly correlated with sarcopenia in individuals aged 40 years or older (*P* trend for non-linear 0.3516, 0.2181, and 0.756, respectively; Figures 4A–C).

**Table 3:** Associations of OBS, dietary OBS, and lifestyle OBS with sarcopenia in people ≥40 years of age with MetS.

**Variables**	**OR (95%CI) *P*-value**	**OR (95%CI) *P*-value**	**OR (95%CI) *P*-value**
**OBS**	0.955 (0.936, 0.974) < 0.0001	0.956 (0.937, 0.976) < 0.0001	0.964(0.942,0.987) 0.003
**OBS quartile**			
Q1	Ref.	Ref.	Ref.
Q2	0.655 (0.458, 0.938) 0.0226	0.651 (0.444, 0.956) 0.0306	0.674 (0.458, 0.991) 0.0472
Q3	0.624 (0.432, 0.900) 0.0130	0.639 (0.434, 0.940) 0.0251	0.685 (0.467, 1.005) 0.0558
Q4	0.440 (0.300, 0.645) 0.0001	0.452 (0.304, 0.673) 0.0002	0.509 (0.324, 0.801) 0.0042
***P*** **for trend**	0.0001	0.0004	0.0060
**OBS. dietary**	0.956 (0.936, 0.977) 0.0001	0.958 (0.937, 0.980) 0.0003	0.967(0.942,0.993) 0.0012
**OBS. dietary quartile**			
Q1	Ref.	Ref.	Ref.
Q2	0.725 (0.495, 1.063) 0.1026	0.718 (0.482, 1.069) 0.1054	0.742 (0.493, 1.119) 0.1576
Q3	0.688 (0.467, 1.012) 0.0603	0.735 (0.490, 1.103) 0.1405	0.784 (0.516, 1.193) 0.2589
Q4	0.436 (0.288, 0.661) 0.0002	0.451 (0.293, 0.694) 0.0005	0.505 (0.306, 0.834) 0.0089
***P*** **for trend**	0.0002	0.0009	0.0162
**OBS. lifestyle**	0.887 (0.813, 0.968) 0.0084	0.877 (0.802, 0.960) 0.0052	0.873(0.797,0.957) 0.0041
**OBS. lifestyle quartile**			
Q1	Ref.	Ref.	Ref.
Q2	0.784 (0.526, 1.170) 0.2367	0.802 (0.538, 1.195) 0.2804	0.774 (0.521, 1.149) 0.2069
Q3	0.833 (0.562, 1.235) 0.3658	0.860 (0.566, 1.308) 0.4832	0.841 (0.554, 1.276) 0.4171
Q4	0.620 (0.425, 0.905) 0.0147	0.588 (0.395, 0.875) 0.0101	0.576 (0.387, 0.856) 0.0074
***P*** **for trend**	0.0260	0.0185	0.0156

MetS, Metabolic Syndrome; OBS, oxidative balance score; PIR, income-poverty ratio.

Crude models did not adjust for any covariates, model 1 adjusted for age, sex, race, PIR, educational attainment, and marital status, and model 2 additionally adjusted for dietary energy intake above model.

### 3.5 Sensitivity analysis

In the sensitivity analysis, we found that similar results were obtained using the IDF criteria for diagnosing MetS, indicating the stability of the conclusions. After controlling for potential confounders, OBS, dietary OBS, and lifestyle OBS each showed an inverse relationship with the incidence of sarcopenia within the MetS demographic [OBS: OR = 0.959, 95% CI: (0.940, 0.979), *P* trend = 0.0001; dietary OBS: OR = 0.967, 95% CI: (0.945, 0.989), *P* trend = 0.0039; lifestyle OBS: OR = 0.828, 95% CI: (0.760, 0.902), *P* trend < 0.0001]. Elevated levels of OBS, dietary OBS, and lifestyle OBS were linked to a substantially reduced occurrence of sarcopenia (all *P* for trend < 0.05; Supplementary Table S3).

## 4 Discussion

In a nationwide, population-based sectional analysis, our findings demonstrated for the first time that a dietary and lifestyle-based oxidative balance metric, OBS, was inversely correlated with sarcopenia among the MetS population, exhibiting dose-response relationships. In the fully adjusted model, for every 1-unit increase in OBS, the risk of sarcopenia in MetS patients was reduced by 4.1% (OR = 0.959, 95%CI: 0.938–0.981, *P* = 0.0005). Compared to Q1, participants with OBS at Q3 and Q4 had a significantly lower prevalence of sarcopenia (OR 0.659 and 0.494, respectively; p for trend = 0.026). Age emerged as a critical modifier in these correlations, manifesting only in individuals aged 40 years and above (OR = 0.964, P = 0.003), while there was no link between OBS and early-onset sarcopenia within the MetS demographic (*P* > 0.05). These results indicate that managing external oxidative stress may play a crucial role in the development of sarcopenia in people with MetS, highlighting the importance of monitoring both dietary and lifestyle sources of antioxidants and pro-oxidants in medical settings. Our research underlines that maintaining a diet and lifestyle rich in antioxidants, evaluated through OBS, is instrumental in preventing sarcopenia among those with MetS, particularly in older adults.

OBS effectively gauges a person's external oxidative equilibrium by encompassing dietary and lifestyle interactions with well-known pro-oxidants and antioxidants. Since the initial proposition of OBS (involving intake assessments of dietary vitamin C, beta carotene, and iron), ongoing studies have refined the components and methodologies for OBS evaluation (32). Substantial clinical studies have proposed their respective components and assessment criteria for OBS, and more than 20 types of OBS are currently represented (20). In this investigation, we utilized the OBS computation method previously adopted in studies leveraging the NHANES dataset, incorporating 16 dietary OBS components and 4 prevalent lifestyle OBS elements (8, 21, 23, 24, 33). Extensive practical research has shown that OBS is linked with the onset and prognosis of various conditions, encompassing a range of cardiometabolic diseases such as cardiovascular disease (CVD) (34), non-alcoholic fatty liver disease (NAFLD) (33), chronic kidney disease (21), metabolic syndrome (MetS) (24), and type 2 diabetes mellitus (35). Additionally, multiple studies have explored the relationship between OBS and sarcopenia across the general populace. A recent case-control study from Iran revealed that OBS significantly inversely correlated with the probability of sarcopenia in older adults (22). A sectional analysis utilizing NHANES data from 2011 to 2018 also demonstrated a negative correlation between OBS and sarcopenia in adults aged 20–59 years [OR = 0.942, 95% CI: (0.920, 0.964), *P* trend < 0.001] (23). Another sectional analysis from NHANES indicated that OBS was significantly inversely related to low muscle mass in the middle-aged demographic (OR = 0.96, *P* trend < 0.0001) (21). Our results, for the first time, indicate that OBS, dietary OBS, and lifestyle OBS all inversely relate to sarcopenia in the MetS group, offering the latest population-level clinical evidence supporting the benefit of higher OBS for preventing sarcopenia in individuals with MetS. It is important to note that previous investigations employing NHANES data from only 2011–2018 (during which DXA scans were performed on individuals aged 8–59 years) imply that their findings were applicable primarily to the non-elderly population (under 60 years). Our study, on the other hand, comprehensively included data from NHANES 1999–2006 and 2011–2018, providing a more comprehensive age range of participants and making the conclusions more generalizable. Interestingly, whereas a previous study showed a non-linear correlation between OBS and sarcopenia in the general population aged 20–59 years (21), our RCS findings illustrate a dose-response relationship between all OBS and sarcopenia in the MetS population.

Mounting research indicates that consuming certain antioxidants can help prevent sarcopenia (36). Foods rich in antioxidants like dietary fiber, micronutrients, vitamins, and polyphenols are beneficial in managing sarcopenia (34, 37). Regular consumption of antioxidant-rich vitamins such as C and E is crucial for neutralizing reactive oxygen species and correcting cellular redox imbalances, potentially reducing sarcopenia by alleviating oxidative stress (33). The role of dietary antioxidants in the diet or as supplements is increasingly recognized as a significant area for sarcopenia research, though definitive benefits in sarcopenia are yet to be established (38). Beyond diet, lifestyle factors that influence antioxidant and pro-oxidant levels also significantly impact sarcopenia progression. Numerous epidemiological studies have identified sedentary lifestyles as primary contributors to sarcopenia. Engaging in suitable physical activities is essential for preventing sarcopenia and enhancing functional outcomes and life quality in affected individuals (39). Several observational studies have noted that excessive alcohol consumption might correlate with lower muscle mass and decreased grip strength, although results are not consistent (40, 41). The link between BMI and sarcopenia also remains debated (42, 43). Moreover, smoking is a major risk factor for developing sarcopenia, yet the relationship between serum cotinine levels, a marker for smoking exposure, and sarcopenia has not been extensively explored (44). While individual antioxidants or pro-oxidants have been identified as potentially relevant to sarcopenia development, research into how redox balance within one's diet and lifestyle affects sarcopenia is still limited. Our research indicates that the OBS, a comprehensive external oxidative stress assessment tool, correlates with sarcopenia in MetS individuals and could inform future prevention strategies.

Oxidative stress is a shared pathophysiological hallmark of MetS and sarcopenia and has an important role in disease development (16, 19). Individuals with comorbid sarcopenia tend to have poorer clinical outcomes than those with MetS alone. A prospective cohort study that included 749 patients with resectable gastric cancer demonstrated a worse prognosis in populations with comorbid preoperative MetS and sarcopenia regardless of whether they were compared to normal, MetS-alone, or sarcopenia-alone patients (17). A recent longitudinal study utilizing NHANES data revealed that the simultaneous presence of MetS and sarcopenia correlated with significantly elevated risks of all-cause and CVD mortality within the general population (45). Therefore, not only do MetS and sarcopenia share potential biological and clinical connections, but the dual occurrence of sarcopenia in those with MetS may profoundly impact clinical outcomes and escalate overall health burdens, particularly as sarcopenia might also contribute to other health issues, including CVD. Identifying risk determinants for sarcopenia in individuals with MetS could aid in its prevention, thus enhancing overall public health outcomes, with our results supporting the beneficial role of an antioxidant-enriched diet and lifestyle in mitigating sarcopenia.

Accumulating experimental evidence suggests that oxidative stress has a key role in the pathogenesis of sarcopenia. Excessive reactive oxygen species (ROS) have important effects on the regulation of muscle protein synthesis and catabolism and on mitochondrial function (46). ROS can directly damage mitochondrial DNA and weaken mitochondrial function, leading to impaired energy metabolism and functional decline in muscle cells (47). Oxidative stress also inhibits the expression of genes involved in mitochondrial biosynthesis, which reduces mitochondrial quantity and quality and exacerbates the decline in muscle function (48). Notably, oxidative stress can crosstalk with inflammatory signaling pathways, which in turn promotes the production and release of multiple inflammatory factors, disrupting the dynamic balance of muscle proteins and accelerating muscle atrophy (49). Interestingly, oxidative stress also affects the structure and function of the neuromuscular junction, depriving the muscle of innervation and consequently atrophy (50). In summary, oxidative stress affects the structure and function of muscle tissue through multiple pathways and is one of the key factors in the pathogenesis of sarcopenia.

Several observational clinical studies have shown that higher OBS is associated with lower levels of oxidative stress markers in individuals. A cross-sectional analysis from Korea showed that OBS was negatively associated with serum γ-glutamyltransferase levels among adults (51). Another cross-sectional analysis from Japan showed that OBS was negatively associated with levels of an oxidative stress marker, urinary 8-hydroxydeoxyguanosine, in adults (52). In a retrospective study from the US, Kong et al. showed that OBS was negatively associated with levels of oxidative stress markers (F2-isoprostanes) and systemic inflammatory markers [C-reactive protein (CRP)] in individuals (53). Another cross-sectional analysis similarly showed that higher OBS was associated with lower serum high-sensitivity-CRP levels and reduced systemic inflammation in middle-aged and older Japanese (54). These significant associations suggest that OBS may modulate the body's intrinsic levels of oxidative stress and inflammation, which may partially explain the negative correlation between OBS and sarcopenia.

Age markedly affected the link between OBS and sarcopenia among MetS patients, with the advantageous impact of OBS on this condition manifesting only in individuals aged 40 and above, suggesting no protective benefit against sarcopenia emerging at younger ages. Sarcopenia, characterized by an age-related gradual decline in muscle mass and function, remains under-researched in younger demographics. Earlier NHANES-based research indicated that factors like obstructive sleep apnea and intake levels of dietary vitamins B1 and B2 are positively and inversely related to early-onset sarcopenia, respectively (30, 55). Recent epidemiological research indicates that sarcopenia might affect one in 10 young adults, potentially linked to MetS, lack of physical activity, genetic and prenatal influences, vitamin D deficiency, hormonal imbalances, and other variables (9). Although current studies do not delve into the foundational mechanisms, our results imply that prevention strategies for MetS-related early-onset sarcopenia may not derive substantial benefits from an antioxidant-focused diet and lifestyle. Further exploration and intervention in other modifiable risk elements are necessary for this specific group.

Our research possesses several notable strengths. It is a comprehensive, population-wide study with a robust sample size and diverse ethnic participation, which enhances the extrapolation of the findings. OBS serves as an integrated measure of oxidative balance, previously well-validated in NHANES-affiliated research, and it offers a more precise reflection of a person's total exposure compared to isolated antioxidants.

### 4.1 Limitations

Nonetheless, our study faces certain limitations. Firstly, its cross-sectional nature precludes causal inference and may be vulnerable to underlying confounding. The cross-sectional design prevents us from determining the temporal sequence of the relationship between OBS and sarcopenia. Prospective detailed cohort studies are essential to confirm the long-term relationships of OBS with sarcopenia in the MetS group. In addition, intervention studies could be designed to clarify potential causal associations. Secondly, while the sarcopenia diagnosis aligns with prior NHANES studies and FNIH standards, it did not permit evaluation of muscle functionality, such as strength or mobility, due to data limitations within NHANES. Further validation of these findings using well-supported diagnostic criteria for sarcopenia is needed. Moreover, the roles of additional factors such as genetic influences, prenatal conditions, and hormonal disorders require further clarification. Although we used an OBS assessment method validated in NHANES, differences in OBS components across studies may lead to limited extrapolation of results and warrant standardization of scoring systems. Assessment of OBS components relies mostly on self-report and may be affected by recall bias; future studies may use more objective assessment instruments to improve the accuracy of OBS scale.

## 5 Conclusions

In a nationwide sectional study, OBS, dietary OBS, and lifestyle OBS each demonstrated significant inverse correlations with sarcopenia prevalence among those with MetS, exhibiting dose-response relationships. Our fully adjusted regression data suggest that each point increase in OBS may reduce the prevalence of sarcopenia in the MetS population by 4.1%, and that OBS in the highest quartile (compared to Q1) may have prevented 50.6% of sarcopenia occurrences. Age markedly impacted these relationships, with the beneficial effects of OBS manifesting in individuals aged 40 years and older. Therefore, we suggest that sarcopenia prevention strategies for patients with MetS should begin in middle age (≥40 years) to enhance OBS scores by improving diet (e.g., increasing antioxidant nutrient intake) and lifestyle (e.g., increasing physical activity, quitting smoking and restricting alcohol). These outcomes indicate that an antioxidant-rich diet and lifestyle in line with OBS metrics might aid in preventing sarcopenia within the MetS demographic, underscoring the need for future rigorously designed longitudinal cohort studies.

## Data Availability

Publicly available datasets were analyzed in this study. This data can be found at: https://www.cdc.gov/nchs/nhanes/.
