# Supplementary material for: Oxidative balance score is independently associated with reduced prevalence of sarcopenia among US adults with metabolic syndrome
Source: Front Nutr. 2025 Apr 8;12:1529140. doi: 10.3389/fnut.2025.1529140 (PMC12011616; doi:10.3389/fnut.2025.1529140)
Supplement: Supplementary file 1 [file Table_1.docx]

Supplementary Material

**Table S1.** Assessment and scoring scheme for OBS, NHANES1999-2006 and 2011-2018.

|  |  | Male |  |  | Female |  |  |
| --- | --- | --- | --- | --- | --- | --- | --- |
|  | Property | 0 | 1 | 2 | 0 | 1 | 2 |
| **Dietary OBS components** |  |  |  |  |  |  |  |
| Dietary fiber (g/d) | A | <12.56 | 12.5-19.67 | >19.67 | <10.05 | 10.05-16.30 | >16.30 |
| Carotene (RE/d) | A | <98.54 | 98.62-306.04 | >306.04 | <98.00 | 98.06-383.35 | >383.35 |
| Riboflavin (mg/d) | A | <1.79 | 1.79-2.69 | >2.69 | <1.34 | 1.34-2.02 | >2.02 |
| Niacin (mg/d) | A | <20.63 | 20.63-29.71 | >29.71 | <14.52 | 14.52-21.85 | >21.85 |
| Vitamin B6 (mg/d) | A | <1.59 | 1.59-2.40 | >2.40 | <1.13 | 1.13-1.77 | >1.77 |
| Total folate (mcg/d) | A | <315.52 | 315.52-491.50 | >491.50 | <250.00 | 250.00-388.50 | >388.50 |
| Vitamin B12 (mcg/d) | A | <3.35 | 3.35-6.20 | >6.20 | <2.21 | 2.21-4.21 | >4.21 |
| Vitamin C (mg/d) | A | <42.31 | 42.31-113.20 | >113.20 | <38.00 | 38.00-98.30 | >98.30 |
| Vitamin E (ATE) (mg/d) | A | <5.82 | 5.82-9.41 | >9.41 | <4.51 | 4.51-7.51 | >7.51 |
| Calcium (mg/d) | A | <646.50 | 646.50-1069.00 | >1069.00 | <500.40 | 500.40-848.78 | >848.78 |
| Magnesium (mg/d) | A | <257.00 | 257.00-361.00 | >361.00 | <187.00 | 187.00-283.21 | >283.21 |
| Zinc (mg/d) | A | <9.75 | 9.75-15.08 | >15.08 | <6.73 | 6.73-10.74 | >10.74 |
| Copper (mg/d) | A | <1.12 | 1.12-1.57 | >1.57 | <0.85 | 0.85-1.28 | >1.28 |
| Selenium (mcg/d) | A | <94.94 | 94.94-141.70 | >141.70 | <67.83 | 67.83-99.10 | >99.10 |
| Total fat (g/d) | P | >107.40 | 69.83-107.40 | <69.83 | >75.73 | 51.01-75.73 | <51.01 |
| Iron (mg/d) | P | >19.15 | 9.65-14.31 | <12.88 | >14.32 | 9.65-14.32 | <9.65 |
| **Lifestyle OBS components** |  |  |  |  |  |  |  |
| Physical activity (MET-minute/week) | A | <409.50 | 409.50-1137.73 | >1137.73 | <270.67 | 270.67-840.00 | >840.00 |
| Alcohol (g/d) | P | >30g/day | 0-30g/day | None | >15g/day | 0-15g/day | None |
| Body mass index (kg/m2) | P | >33.77 | 29.37-33.77 | <29.37 | >31.40 | 28.38-31.40 | <28.38 |
| Cotinine (ng/mL) | P | >1.06 | 0.04-1.06 | <0.04 | >0.18 | 0.04-0.18 | <0.04 |

A: antioxidant; P: pro-oxidant；MetS, Metabolic Syndrome; OBS, oxidative balance score.

**Table S2**. Association of all OBS with early-onset sarcopenia in the MetS population.

|  | **OR (95%CI) P-value** | **OR (95%CI) P-value** | **OR (95%CI) P-value** |
| --- | --- | --- | --- |
| **OBS** | 1.005 (0.971, 1.039) 0.7881 | 0.996 (0.963, 1.031) 0.8299 | 1.010(0.948,1.077)  0.759 |
| **OBS quartile** |  |  |  |
| Q1 | Ref. | Ref. | Ref. |
| Q2 | 1.306 (0.524, 3.256) 0.5680 | 1.137 (0.459, 2.817) 0.7822 | 1.189 (0.473, 2.990) 0.7135 |
| Q3 | 0.777 (0.374, 1.614) 0.4998 | 0.712 (0.337, 1.504) 0.3756 | 0.772 (0.350, 1.702) 0.5228 |
| Q4 | 0.987 (0.465, 2.096) 0.9724 | 0.829 (0.391, 1.756) 0.6253 | 0.954 (0.409, 2.226) 0.9136 |
| **P for trend** | 0.6287 | 0.3816 | 0.6543 |
| **OBS. dietary** | 1.001 (0.967, 1.036) 0.9562 | 0.994 (0.960, 1.028) 0.7094 | 1.007(0.940,1.078)  0.840 |
| **OBS. dietary quartile** |  |  |  |
| Q1 | Ref. | Ref. | Ref. |
| Q2 | 0.714 (0.275, 1.856) 0.4909 | 0.636 (0.244, 1.657) 0.3569 | 0.662 (0.247, 1.775) 0.4146 |
| Q3 | 0.885 (0.402, 1.951) 0.7629 | 0.804 (0.359, 1.801) 0.5966 | 0.871 (0.378, 2.004) 0.7456 |
| Q4 | 0.807 (0.380, 1.713) 0.5778 | 0.692 (0.328, 1.463) 0.3373 | 0.791 (0.333, 1.877) 0.5955 |
| **P for trend** | 0.7365 | 0.5005 | 0.7968 |
| **OBS. lifestyle** | 1.108 (0.887, 1.384) 0.3661 | 1.064 (0.856, 1.321) 0.5781 | 1.091(0.869,1.371)  0.4487 |
| **OBS. lifestyle quartile** |  |  |  |
| Q1 | Ref. | Ref. | Ref. |
| Q2 | 0.988 (0.411, 2.379) 0.9791 | 0.992 (0.406, 2.425) 0.9868 | 0.949 (0.384, 2.343) 0.9095 |
| Q3 | 0.999 (0.411, 2.426) 0.9975 | 0.961 (0.394, 2.341) 0.9301 | 0.949 (0.388, 2.322) 0.9097 |
| Q4 | 1.803 (0.839, 3.876) 0.1336 | 1.536 (0.711, 3.318) 0.2772 | 1.459 (0.671, 3.170) 0.3424 |
| **P for trend** | 0.2200 | 0.3815 | 0.4347 |

Crude models did not adjust for any covariates, model 1 adjusted for age, sex, race, PIR, educational attainment, and marital status, and model 2 additionally adjusted for dietary energy intake above model 1.

MetS, Metabolic Syndrome; OBS, oxidative balance score; PIR, income-poverty ratio.

**Table S3**. Association of OBS with prevalence of sarcopenia in the MetS population diagnosed by IDF criteria.

|  | **Crude Model**  **OR (95%CI) P-value** | **Model 1**  **OR (95%CI) P-value** | **Model 2**  **OR (95%CI) P-value** |
| --- | --- | --- | --- |
| **OBS** | 0.961 (0.947, 0.976) <0.0001 | 0.963 (0.948, 0.978) <0.0001 | 0.959 (0.940, 0.979) 0.0001 |
| **OBS quartile** |  |  |  |
| Q1 | Ref. | Ref. | Ref. |
| Q2 | 0.793 (0.573, 1.097) 0.1640 | 0.768 (0.546, 1.080) 0.1315 | 0.763 (0.540, 1.078) 0.1277 |
| Q3 | 0.607 (0.448, 0.822) 0.0016 | 0.637 (0.461, 0.880) 0.0073 | 0.629 (0.443, 0.895) 0.0112 |
| Q4 | 0.502 (0.367, 0.686) <0.0001 | 0.518 (0.378, 0.710) 0.0001 | 0.507 (0.343, 0.751) 0.0010 |
| **P for trend** | <0.0001 | <0.0001 | 0.0006 |
| **OBS. dietary** | 0.965 (0.949, 0.981) <0.0001 | 0.968 (0.951, 0.984) 0.0002 | 0.967 (0.945, 0.989) 0.0039 |
| **OBS. dietary quartile** |  |  |  |
| Q1 | Ref. | Ref. | Ref. |
| Q2 | 0.733 (0.518, 1.039) 0.0834 | 0.709 (0.495, 1.016) 0.0636 | 0.711 (0.486, 1.040) 0.0819 |
| Q3 | 0.722 (0.517, 1.008) 0.0584 | 0.764 (0.535, 1.092) 0.1429 | 0.768 (0.527, 1.118) 0.1717 |
| Q4 | 0.505 (0.361, 0.707) 0.0001 | 0.526 (0.374, 0.741) 0.0004 | 0.531 (0.343, 0.821) 0.0052 |
| **P for trend** | 0.0002 | 0.0010 | 0.0099 |
| **OBS. lifestyle** | 0.846 (0.780, 0.919) 0.0001 | 0.830 (0.762, 0.905) <0.0001 | 0.828 (0.760, 0.902) <0.0001 |
| **OBS. lifestyle quartile** |  |  |  |
| Q1 | Ref. | Ref. | Ref. |
| Q2 | 0.744 (0.517, 1.070) 0.1135 | 0.745 (0.516, 1.075) 0.1184 | 0.728 (0.505, 1.050) 0.0920 |
| Q3 | 0.676 (0.471, 0.969) 0.0352 | 0.661 (0.450, 0.972) 0.0375 | 0.654 (0.446, 0.959) 0.0321 |
| Q4 | 0.566 (0.404, 0.792) 0.0012 | 0.530 (0.373, 0.753) 0.0006 | 0.524 (0.369, 0.743) 0.0005 |
| **P for trend** | 0.0014 | 0.0008 | 0.0007 |

Crude models did not adjust for any covariates, model 1 adjusted for age, sex, race, PIR, educational attainment, and marital status, and model 2 additionally adjusted for dietary energy intake above model 1.

MetS, Metabolic Syndrome; OBS, oxidative balance score; PIR, income-poverty ratio.
